# Get a better glimpse on sequential photoreactions of trisnorbornadienes with ^19^F NMR spectroscopy

**DOI:** 10.3762/bjoc.22.38

**Published:** 2026-03-23

**Authors:** Julian Felix Maria Hebborn, Ben Eric Merten, Thomas Paululat, Heiko Ihmels

**Affiliations:** 1 Department of Chemistry-Biology, and Research Center of Micro- and Nanochemistry and (Bio-)Technology (Cμ), University of Siegen, Adolf-Reichwein-Str. 2, 57068 Siegen, Germany

**Keywords:** fluoroarenes, molecular solar thermal energy storage, photochemistry, photochromism, quadricyclanes

## Abstract

It is shown exemplarily with a trifluorinated trisnorbornadienylbenzene that ^19^F NMR spectroscopy may be applied as a useful complementary method for the investigation of sequential photoreactions. The trisnorbornadiene core structure was used as it figures as promising scaffold for molecular solar thermal (MOST) energy storage. The target compound was readily synthesized by a Suzuki–Miyaura coupling reaction and examined with respect to the key properties for MOST applications. Upon direct or photosensitized irradiation, the trisnorbornadiene was transformed stepwise and almost quantitatively into the corresponding trisquadricyclane. Even though the reaction can be monitored by photometry or by in situ ^1^H NMR spectroscopy, unambiguous assignment of the distinct intermediate mono- and bisquadricyclanes was not possible because of signal overlap. In contrast, this shortcoming is circumvented with in situ ^19^F NMR-spectroscopic analysis. Contrary to the ^1^H NMR spectra, the ^19^F NMR spectra show significantly fewer characteristic and sufficiently separated signals that allow the unambiguous identification of all photoproducts and, thus, their detection in the course of the photoreaction.

## Introduction

**Scheme 1:**
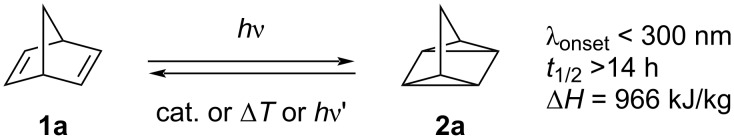
Photochromic reaction of norbornadiene (**1a**) and quadricyclane (**2a**) and selected physicochemical properties; λ_onset_ = low-energy zero onset of absorption of **1a**; *t*_1/2_ = half-life of **2a**; Δ*H* = reaction enthalpy of the cycloreversion of **2a**.

Photochromic compounds, which change their physical and chemical properties reversibly upon irradiation, figure as a versatile basis for the development of functional materials, whose performance can be switched or controlled by light [1–4]. To add to that, the photochromic reaction allows to convert the energy of the applied light into chemical energy, which can thus be stored and eventually released on demand as heat in the back reaction. Therefore, photochromic reactions constitute the centerpiece of the emerging approach towards molecular solar thermal (MOST) energy storage [5–8]. In this context, norbornadienes are employed as well established photoswitches because they provide several favorable photochemical and physicochemical properties [9–10]. In a photochromic intramolecular [2 + 2] cycloaddition reaction, these compounds are transformed into an energy-rich metastable photoproduct that, upon application of an external stimulus, isomerizes back in a cycloreversion reaction to the starting compound with the release of heat (Scheme 1) [5,7]. In comparison with other photochromic compounds (e.g., azobenzenes [11–12], anthracenes [13–15], stilbenes [16–17], and bicyclooctadienes [18–19]), the photoisomers of norbornadienes, namely quadricyclanes, stand out with their high energy density, with the parent compound **2a** providing a Δ*H* value of 966 kJ/kg [20–23] and a half-life of the more than 14 h [24]. At the same time, however, norbornadiene (**1a**) has also some major drawbacks with regard to a real MOST application, namely minimal overlap of the absorption spectrum (λ_onset_ < 300 nm) [25–28] with the solar emission spectrum as well as a low quantum yield (Ф = 5%) [26–27].

**Figure 1:**
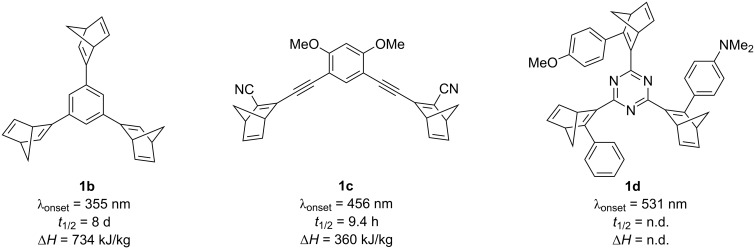
Structures and MOST parameters of representative bis- and trisnorbornadienes **1b**–**d**; *t*_1/2_ = half-life determined at 25 °C; n.d. = not determined.

In order to optimize the photophysical and physicochemical properties of norbornadiene (**1a**) with minimal impact on the energy density, scaffolds with multiple norbornadiene units have been investigated with the goal to accomplish higher energy storage density per molecule [20–22,29–33]. Furthermore, to shift the absorption towards lower-energy light in such compounds – and for that matter to the solar spectrum – push–pull systems [29,31], enlarged π systems [30,33], and heteroaromatic chromophores [32,34–35] have been introduced (Figure 1). Although this structural variation often leads to a red-shifted absorption, the half-lives of the corresponding quadricyclanes are often decreased at the same time. In particular, the addition of an acetylene unit between the linking aromatic part and the norbornadiene often leads to short half-lives [29–31].

As arene-linked bis- and trisnorbornadienes are promising lead structures for further development of more efficient MOST materials, a detailed investigation of the photochemical reactions is necessary. However, such studies of multichromophore photoreactions are often difficult, because the distinct reaction steps are not necessarily consecutive and may not be readily dissected. Furthermore, as the reaction intermediates are isomers with mainly similar structural fragments, it is difficult to distinguish them by the usually employed spectroscopic methods because of overlapping signals [22,30,33]. Along these lines, ^1^H NMR spectroscopy is a very useful method to follow the photoreaction, ideally upon direct irradiation in the NMR probehead (in situ NMR), because it potentially enables accurate structure elucidation. But even though this method may enable the accurate monitoring of a stepwise photochromic reaction of multichromophores in some cases [36–37], significant overlap of NMR signals often interferes with the detailed identification of separate components. Therefore, we proposed that the analysis of the photoreaction of a fluorinated starting material with in situ ^19^F NMR spectroscopy may add a useful complementary tool [38–41]. Firstly, the ^19^F nucleus is only slightly less sensitive for NMR spectroscopy than the ^1^H nucleus [42]. Additionally, fluoro substituents may be attached such that they will give well-separated signals in less complex spectra, thus allowing conclusive and quantitative product identification [38–40]. Herein, we will show in a proof-of-principle study with a fluorinated trisnorbornadiene derived from promising lead compound **1b** that in situ ^19^F NMR spectroscopy may indeed be used to monitor sequential photoreactions of multichromophoric systems.

## Results

**Scheme 2:**
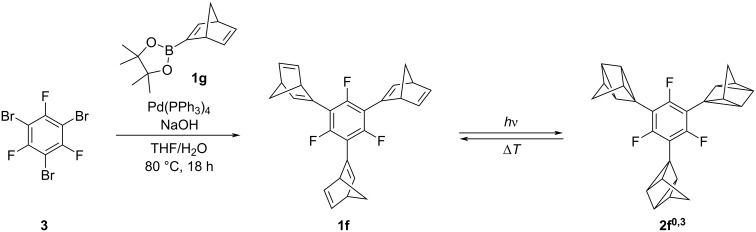
Synthesis and photochromic reaction of trisnorbornadiene **1f** to the trisquadricyclane **2f^0,3^** (numbering of quadricyclanes **2f*^m^*^,^*^n^***: *m* = number of norbornadiene units, *n* = number of quadricyclane units).

The trisnorbornadiene **1f** was synthesized in 36% yield through a Suzuki–Miyaura coupling reaction of tribromotrifluorobenzene **3** with the 2-borononorbornadiene **1e** (Scheme 2) [43]. The product **1f** was identified and fully characterized by NMR spectroscopy (^1^H, ^13^C, ^19^F, COSY, HSQC, HMBC), mass spectrometry, melting point, and elemental analysis. Norbornadiene **1f** is well soluble in alkanes, chlorinated and aromatic solvents, whereas it is hardly soluble in polar protic solvents and MeCN and insoluble in water.

The absorption spectra of **1f** were recorded in MeCN, cyclohexane, MeOH, ethyl acetate, THF, CH_2_Cl_2_, CHCl_3_, and benzene (see Supporting Information File 1, Figure S1). The absorption spectra have essentially the same key features, specifically absorption maxima at around 209 nm, 240 nm, and 273 nm, with a long-wavelength absorption onset at 350 nm. Both maxima and onset absorptions are hypsochromically shifted compared with the ones of the parent compound **1b** [22].

**Figure 2:**
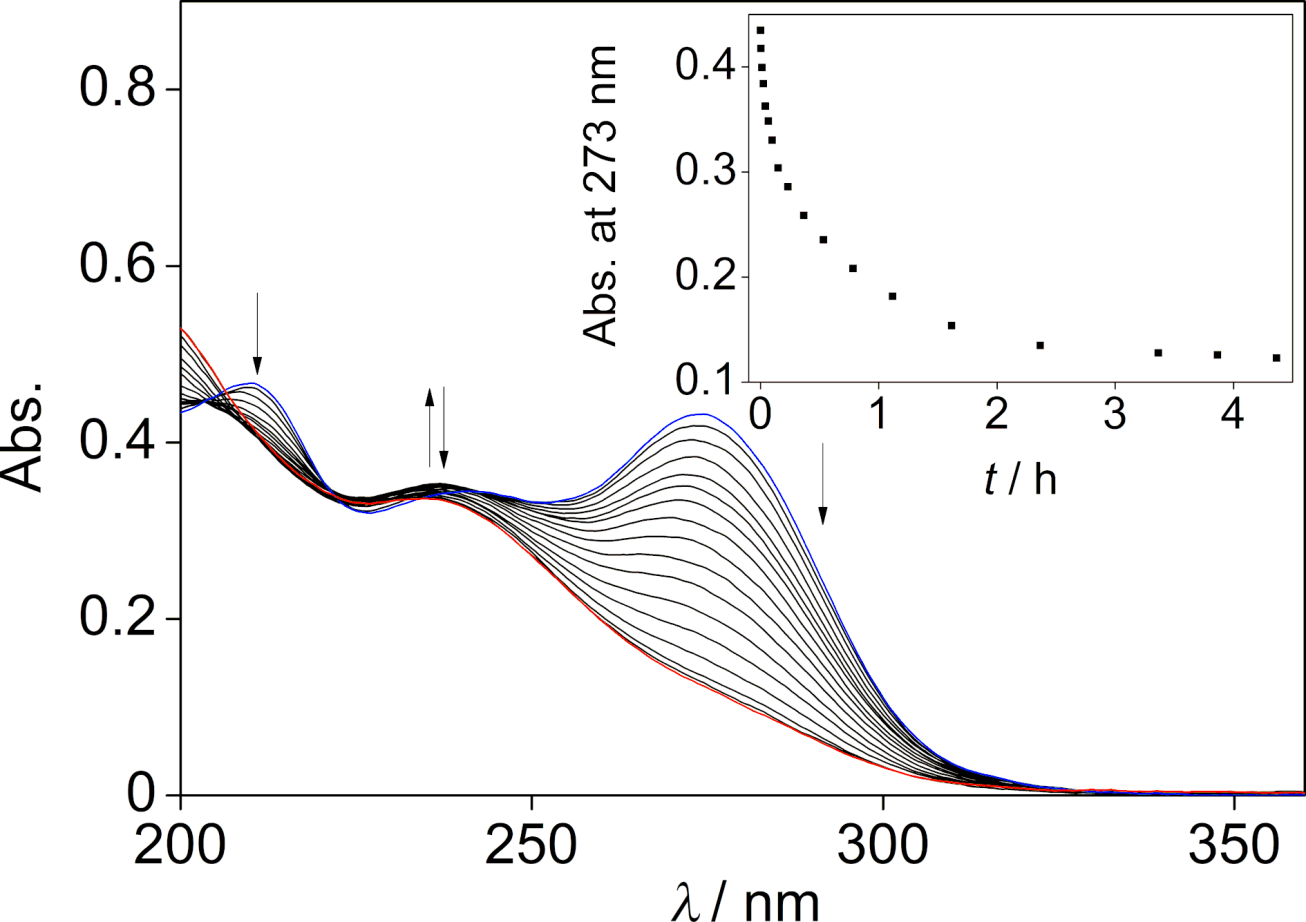
Photometric monitoring of the photoreaction of **1f** in MeCN (*c* = 20 µM) at λ_ex_ = 315 nm. Inset: plot of absorption at 273 nm versus reaction time.

The [2 + 2] photocycloaddition reaction of **1f** was initiated by direct irradiation with 315 nm. The photoreaction was significantly slower (4 h to achieve photostationary state) compared with the one of the parent compound **1b** (0.5 h) [22]. Monitoring the photoreaction by absorption spectroscopy revealed the formation of a new maximum at 235 nm with no discernible isosbestic points, clearly indicating the presence of more than two differently absorbing compounds (Figure 2). In addition, the examination of the photoreaction with ^1^H NMR spectroscopy showed that the quadricyclane **2f^0,3^** was formed almost quantitatively as the photoproduct (>95%) (see Supporting Information File 1, Figure S11).

**Figure 3:**
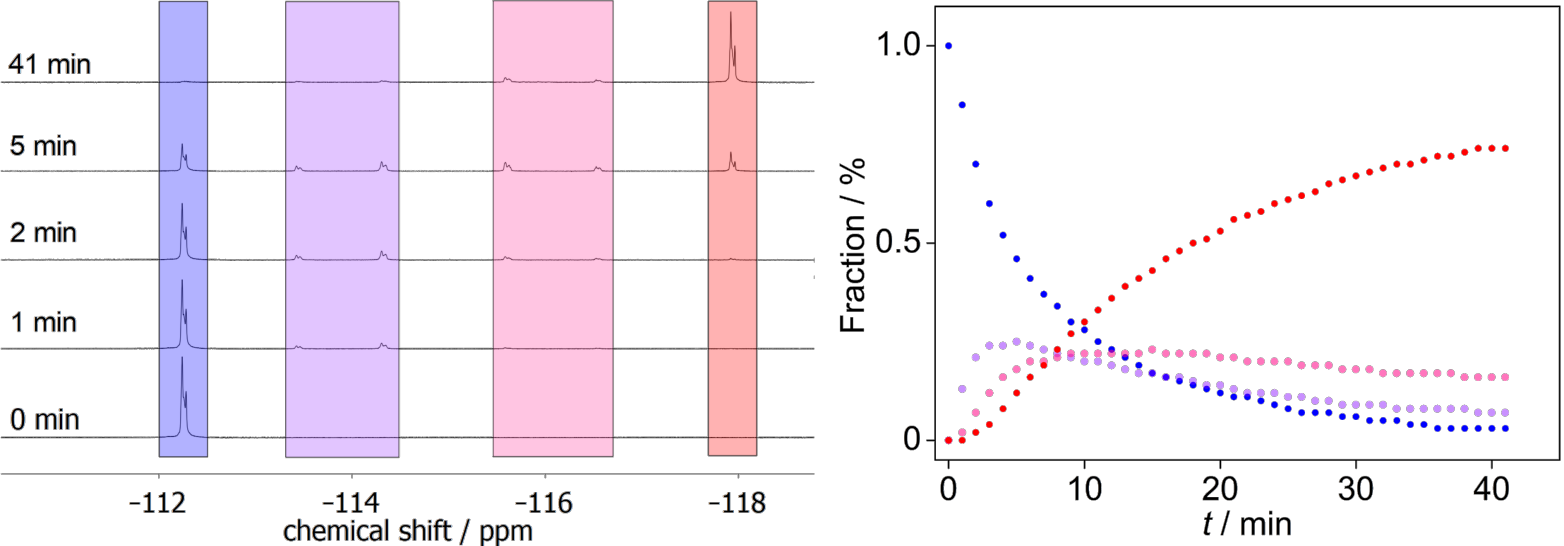
Left: ^19^F NMR-spectroscopic monitoring of the photocycloaddition reaction of **1f** in the presence of flavine **4** in benzene-*d*_6_ (λ_ex_ = 405 nm). Right: Plot of the fraction of components in the reaction mixture during the photoreaction of **1f**; blue: **1f**; purple: **2f^2,1^**; pink: **2f^1,2^**; red: **2f^0,3^**.

**Scheme 3:**
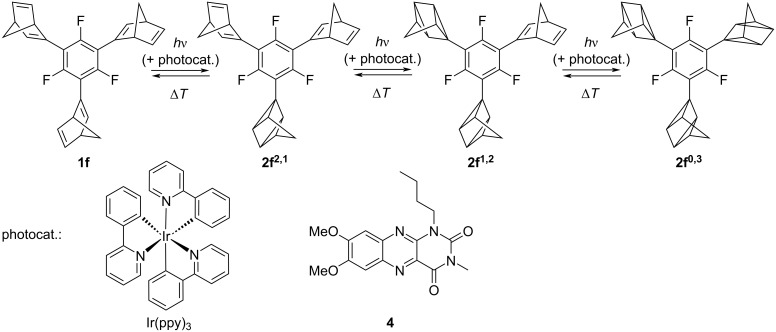
Stepwise photoreaction of **1f** to **2f^0,3^** (numbering of quadricyclanes **2f*^m^*^,^*^n^***: *m* = number of norbornadiene units, *n* = number of quadricyclane units).

The photosensitized reaction in the presence of Ir(ppy)_3_ also afforded quadricyclane **2f^0,3^** in almost quantitative yield (>95%), as shown by ^1^H NMR spectroscopy (see Supporting Information File 1, Figure S10). The photosensitized reaction in the presence of Ir(ppy)_3_ or 1-butyl-7,8-dimethoxy-3-methylalloxazin (**4**) as an alternative catalyst [44–45] with λ_ex_ = 405 nm (LED) was investigated by in situ ^1^H NMR spectroscopy. In both cases, the photoreaction was almost quantitative (see Supporting Information File 1, Figures S13 and S14). To gain more information about the course of the photoreaction and sequential formation of intermediates, the photoreaction of **1f**, catalyzed by flavin **4**, was investigated with in situ ^19^F NMR-spectroscopic analysis. During the irradiation, the NMR signals of two intermediates (approx. −114 ppm and −116 ppm) were observed (Figure 3 and Supporting Information File 1, Figure S15), which were assigned to monoquadricyclane **2f^2,1^** and bisquadricyclane **2f^1,2^** (Scheme 3, Figure 3). As expected, the quadricyclanes were formed consecutively, that is, from monoquadricyclane **2f^2,1^** to bisquadricyclane **2f^1,2^** and eventually the trisquadricyclane **2f^0,3^**. The latter was only formed after a sufficient concentration of the intermediates was present (5 min). From this point onwards, the fraction of the starting material **1f** and the intermediates **2f^2,1^** and **2f^1,2^** decreased steadily, while the content of the trisquadricyclane **2f^0,3^** increased (Figure 3). This proposed consecutive, stepwise photoreaction was supported by theoretical analysis which showed a consistent fitting of the experimental data to the proposed kinetic model (see Supporting Information File 1, Figure S16). Contrary to the ^1^H NMR spectra, the ^19^F NMR spectra showed significantly fewer signals (one or two per molecule) during the photoreaction because of the symmetry of norbornadiene **1f** and its photoproducts and the less complex coupling pattern. Thus, the ^19^F NMR spectra of the norbornadiene **1f** and the quadricyclane **2f^0,3^** showed a multiplet, respectively, whereas the ones of the intermediates resulted in two multiplets with a ratio of 1:2. Additionally, pronounced downfield shifts of the ^19^F NMR signals were observed with increasing number of quadricyclane units (Figure 3).

To assess the physicochemical parameters of the light energy storage and release, the thermally induced cycloreversion of **2f^0,3^** was investigated. Analysis of the cycloreversion at different temperatures revealed a half-life, *t*_1/2_, of 55 d at 25 °C, an activation enthalpy Δ*H* = 99.4 kJ/kg, and an activation entropy of Δ*S* = −42 J/K mol (see Supporting Information File 1, Figure S2).

The energy density of trisquadricyclane **2f^0,3^** was determined with simultaneous thermal analysis (STA) with a heat release of 458 kJ/kg. Additionally, a second exothermic peak (>150 °C) correlated with a significant decrease in mass, indicating decomposition above this temperature (see Supporting Information File 1, Figure S3).

The cycloreversion of trisquadricyclane **2f^0,3^** was also initiated in a ground-state reaction with magic blue (**5**), which has already been shown to be an effective catalyst for this reaction [46–48]. Upon addition of magic blue (**5**, 7.5 mol %) to a solution of quadricyclane **2f^0,3^** in CDCl_3_, the norbornadiene **1f** was formed almost quantitatively in addition to very small traces of tri(4-bromophenyl)amine, i.e., the reduced catalyst (see Supporting Information File 1, Figure S12).

## Discussion

A comparison of the synthesis and photophysical properties of the fluoro-substituted norbornadiene **1f** with the ones of the parent compound **1b** revealed that the yield of the Suzuki–Miyaura coupling reaction is significantly lower for the former [22], presumably because of the steric hindrance induced by the *ortho*-fluoro substituents. These substituents also induce a strong electron-withdrawing effect on the chromophore and thus lower the HOMO energy, likely leading to a larger HOMO–LUMO gap and to a small hypsochromic shift of the absorption (**1f**: λ_max_ = 273 nm; parent norbornadiene **1b**: λ_max_ = 283 nm) [49].

According to the results of the photosensitized reaction, the triplet state energy of the norbornadiene **1f** is higher than that of norbornadiene **1b** (2.08 eV) [50], as the sensitized photocycloaddition only took place with the photocatalyst Ir(ppy)_3_ (2.52 eV) [51] and with flavine (2.28 eV) [52]. In contrast, [Ru(phen)_3_]^2+^ (2.19 eV) [53] does not catalyze the reaction of **1f**, but is still able to catalyze the one of **1b** [50].

Notably, the half-life of trisquadricyclane **2f^0,3^** (55 d) is almost eight times longer than the one of the parent compound **2b** (8 d) [22], presumably because of additional steric hindrance induced by the fluoro substituents in the *ortho* positions [54]. It should be noted that there is only very limited data available to compare the effect of the substitution pattern on the half-life of aryl-substituted norbornadienes to support this interpretation [55]. However, the effect of *ortho* substituents has been examined in more detail for 2-cyano-3-arylnorbornadienes. And these studies have shown that the substituents in *ortho* position lead to an increased thermal stability in comparison with the *meta* and *para*-substituted isomers [54]. Although the longer half-life of the trisquadricyclane may be considered an improved property, the energy density of this compound is lower than that of the parent compound **2b** because of the higher molecular mass.

The experimental results showed a decomposition of trisquadricyclane **2f^0,3^** at higher temperature, which is accompanied by significant loss of mass. This observation likely indicates the thermally induced retro-Diels–Alder reaction of the initially formed norbornadiene units and presumably subsequent reactions of the primary intermediates under these conditions. This unfavorable property may also be caused by the *ortho*-fluoro substituents as it was not observed with the parent compound **1b**. In fact, increased rates of retro-Diels–Alder reactions have been observed with acceptor-substituted substrates [56–57].

Although the course of the photoreaction of norbornadiene **1f** can be visualized by in situ ^1^H NMR spectroscopy, significantly overlapping signals prevent the conclusive differentiation of the individual species that are formed throughout the reaction (see Supporting Information File 1, Figures S13 and S14), interfering with the complete assessment of this stepwise photoreaction. In contrast, it is demonstrated herein that this problem is circumvented by the application of in situ ^19^F NMR-spectroscopic measurements. Contrary to the ^1^H NMR spectra, the ^19^F NMR spectra are less complex and show characteristic and sufficiently separated signals that allow the unambiguous identification of photoproducts **2f^0,3^**, **2f^2,1^**, and **2f^1,2^** and, thus, their detection in the course of the photoreaction.

## Conclusion

One ongoing problem during assessment of MOST systems is the examination of multichromophore photoreactions, because the intermediates share high structural similarities and are thus often difficult to be distinguished by ^1^H NMR spectroscopy, i.e., the currently most applied method. We demonstrated in this proof-of-principle study that fluorinated norbornadiene **1f** is a well-designed substrate for in situ ^19^F NMR-spectroscopic investigation. The advantages of ^19^F NMR spectroscopy are obvious, as the ^19^F NMR spectra comprise fewer and sufficiently separated signals as compared with the ^1^H NMR spectra. To add to that, in principle only one well-placed fluoro substituent is necessary to take advantage of ^19^F NMR spectroscopy as a useful complementary method for the investigation of sequential photoreactions.

At the same time, the norbornadiene/quadricyclane system **1f**/**2f^0,3^** has some favorable MOST properties, namely a high energy density and a long half-life of the trisquadricyclane. In addition, the cycloaddition reaction can be initiated under mild conditions with visible light (λ_ex_ = 405 or 420 nm) in the presence of Ir(ppy)_3_ or flavine **4** as photocatalysts. However, these key parameters do not match the better ones of the parent norbornadiene **1b**. Furthermore, the fluoro-substituted derivative **2f^0,3^** appears to be prone towards thermal decomposition. Thus, even though this compound serves as an ideal probe for the investigation of the photoreaction by ^19^F NMR spectroscopy, further structural optimization is necessary for application purposes.

## Experimental

### Synthesis

#### 1,3,5-Tris(bicyclo[2.2.1]hepta-2,5-dien-2-yl)-2,4,6-trifluorobenzene (**1f**)

A mixture of 1,3,5-tribromo-2,4,6-trifluorobenzene (212 mg, 575 µmol), 4,4,5,5-tetramethyl-2-(bicyclo[2.2.1]heptadien-2-yl)-1,3,2-dioxaborolane (**1e**, 501 mg, 2.30 mmol), Pd(PPh_3_)_4_ (66.5 mg, 57.5 µmol, 5 mol %), THF (5.0 mL), and aq. NaOH (5.7 mmol, 2.7 M, 2.1 mL) was stirred at 80 °C for 16 h under anaerobic conditions [22]. After cooling the emulsion to room temperature, EtOAc (15 mL) was added and the organic layer was separated and dried with Na_2_SO_4_. The drying agent was filtered off, the solvent was removed under reduced pressure, and the crude product was purified by column chromatography (SiO_2_, *n-*hexane, *R*_f_ = 0.44) and recrystallization from *n*-pentane. The product was obtained as colorless cubes (84.0 mg, 209 µmol, 36%); mp 107–109 °C; ^1^H NMR (500 MHz, CDCl_3_) δ 2.08 (dt, ^2^*J* = 6.0 Hz, ^3^*J* = 1.8 Hz, 3H, 7'-H, 7''-H, 7'''-H), 2.19 (dt, ^2^*J* = 6.0 Hz, ^3^*J* = 1.8 Hz, 3H, 7'-H, 7''-H, 7'''-H), 3.71 (br. s, 3H, 4'-H, 4''-H, 4'''-H), 3.89 (br. s, 3H, 1'-H, 1''-H, 1'''-H), 6.82 (dd, ^3^*J* = 5.0 Hz, ^3^*J* = 3.1 Hz, 3H, 5'-H, 5'''-H, 5'''-H), 6.94 (dd, ^3^*J* = 5.0 Hz, ^3^*J* = 2.4 Hz, 3H, 6'-H, 6''-H, 6'''-H), 7.00 (d, ^3^*J* = 2.4 Hz, 3H, 3'-H, 3''-H, 3'''-H); ^13^C NMR (125 MHz, CDCl_3_) δ 50.7 (C4', C4'', C4'''), 54.6 (C1', C1'', C1'''), 73.1 (C7', C7'', C7'''), 111.2 (C1, C3, C5), 143.1 (C5', C5'', C5'''), 143.4 (C6', C6'', C6'''), 144.5 (C3', C3'', C3'''), 144.7 (C2', C2'', C2'''), 154.8 (dt, ^1^*J* = 251.0 Hz, ^3^*J* = 10.9 Hz, C2, C4, C6); ^19^F NMR (470 MHz, CDCl_3_) δ −112.1–112.2 (m, 3F, 2-F, 3-F, 6-F); APCIMS (*m*/*z*): [M + H]^+^ 403; Anal. calcd for C_27_H_21_F_3_: C, 80.58; H, 5.26; found: C, 80.51; H, 5.09.

#### General procedure (GP A) for the sensitized photocycloaddition reaction [48]

A solution of the norbornadiene (4.02–25.0 mg, 10.0–62.1 mmol) and the sensitizer Ir(ppy)_3_ or [Ru(phen)_3_](PF_6_)_2_ (5–10 mol %) in deaerated benzene or MeCN was irradiated with light (λ_ex_ = 420 or 520 nm) for 0.5–3 h. The solvent was removed under reduced pressure at 20 °C and the residue was suspended in *n*-pentane (10 mL) and filtered. The solvent was removed under reduced pressure at 20 °C.

#### 1,3,5-Tris(tetracyclo[3.2.0.0^2,7^.0^4,6^]heptyl)-2,4,6-trifluobenzene (**2f^0,3^**)

Attempt 1: According to GP A, a solution of **1f** (4.02 mg, 10.0 µmol), [Ru(phen)_3_](PF_6_)_2_ (0.47 mg, 0.50 µmol, 5 mol %) in deaerated MeCN (10 mL) was irradiated with λ_ex_ = 520 nm at rt for 30 min. The ^1^H NMR-spectroscopic analysis showed no conversion of the starting material.

Attempt 2: According to GP A, a solution of **1f** (25.0 mg, 62.1 µmol), Ir(ppy)_3_ (4.08 mg, 6.21 µmol, 10 mol %) in deaerated benzene (4 mL) was irradiated with λ_ex_ = 420 nm at rt for 3 h. The product was obtained as an opaque viscous liquid (24.6 mg, 61.1 µmol, >95%). ^1^H NMR (500 MHz, CDCl_3_) δ 1.54–1.57 (m, 1H), 1.67 (ddd, ^3^*J* = 6.0 Hz, ^3^*J* = 4.5 Hz, ^3^*J* = 2.2 Hz, 1H), 1.73–1.78 (m, 1H), 1.84–1.89 (m, 1H), 2.10 (dt, ^2^*J* = 11.1 Hz, ^3^*J* = 1.4 Hz, 1H), 2.21 (dt, ^3^*J* = 4.5 Hz, ^3^*J* = 2.2 Hz, 1H), 2.24 (dt, ^2^*J* = 11.1 Hz, ^3^*J* = 1.4 Hz, 1H).

## Supporting Information

File 1 Experimental section and copies of spectra.

## Data Availability

All data that supports the findings of this study is available in the published article and/or the supporting information of this article.
