# Supplementary material for: Get a better glimpse on sequential photoreactions of trisnorbornadienes with 19F NMR spectroscopy
Source: Beilstein J Org Chem. 2026 Mar 23;22:527–34. doi: 10.3762/bjoc.22.38 (PMC13040267; doi:10.3762/bjoc.22.38)
Supplement: File 1 — Experimental section and copies of spectra. [file Beilstein_J_Org_Chem-22-527-s001.pdf]

BEILSTEIN JOURNAL OF ORGANIC CHEMISTRY

Supporting Information

for

Get a better glimpse on sequential photoreactions of trisnorbornadienes with ^{19}F NMR spectroscopy

Julian Felix Maria Hebborn, Ben Eric Merten, Thomas Paululat and Heiko Ihmels

Beilstein J. Org. Chem. **2026,** *22,* 527–534. doi:10.3762/bjoc.22.38

Experimental section and copies of spectra

License and Terms: This is a supporting information file under the terms of the Creative Commons Attribution License (https://creativecommons.org/licenses/by/4.0). Please note that the reuse, redistribution and reproduction in particular requires that the author(s) and source are credited and that individual graphics may be subject to special legal provisions.
The license is subject to the *Beilstein Journal of Organic Chemistry* terms and conditions: (https://www.beilstein-journals.org/bjoc/terms)

Table of contents

1. Equipment S2
2. Method S2
3. Materials S2
4. Absorption properties S2
5. Photoreactions S3
6. Radical-cation-induced cycloreversion S3
7. Kinetic studies of the thermal back conversion S3
8. STA measurement S4
9. NMR spectra S5
10. In situ NMR spectroscopy S10
11. Kinetic simulation S11
12. References S12

1. Equipment

NMR spectra: JEOL ECZ 500 (^{1}H: 500 MHz, ^{13}C: 125 MHz, ^{19}F: 470 MHz, 25 °C), Varian VNMR-S 600 (^{1}H: 600 MHz, ^{13}C: 150 MHz, ^{19}F: 565 MHz, 25 °C). Reference: residual signals of $CDCl_3$ [$\delta(^{13}C)$ = 77.16 ppm] and C_6D_6 [$\delta(^1H)$ = 7.16 ppm, $\delta(^{13}C)$ = 128.1 ppm], or tetramethylsilane (TMS) [$\delta(^1H)$ = 0.00 ppm], or C_6F_6 [$\delta(^{19}F)$ = −161.6 ppm]. Software: MestReNova. Melting points: Büchi 545 (Büchi, Flawil, CH), uncorrected. Elemental analysis: data in-house (Organic Chemistry, University of Siegen), HEKAtech EUROEA combustion analyzer. Absorption spectra: Varian Cary 100 Bio, Analytik Jena SPECORD S, in Hellma quartz glass cuvettes 115 F-QS (d = 10 mm); Software: Origin (OriginPro 8.5.1) with the implemented smoothing function “adjacent averaging”, factor 10. Photoreactions: 520 nm LED (Conrad electronic Nr. 181862), LUMOS 43 Atlas Photonics; 275 nm, 315 nm, 360 nm, 420 nm). In situ NMR irradiations: 405 nm LEDs [1], the ^{19}F NMR spectra are referenced relative to spectrometer calibration. Simultaneous thermal analysis (STA): STA 449C Jupiter (Netzsch), with platinum crucibles with priced lids in continuous N_2 flow.

2. Methods

Reaction mixtures were stirred with a magnetic stirring bar (400–750 rpm). Solvents were removed with a rotary evaporator at 20–40 °C under reduced pressure (360–15 mbar). Air-sensitive reactions were performed under an inert atmosphere (Ar) with Schlenk equipment. Solvents/solutions were deaerated by bubbling Argon trough the solution (approx. 5 min) prior to use. Room temperature (rt) was between 20 °C and 25 °C.

3. Materials

Commercially available chemicals were purchased from BLD Pharmtech Ltd. (1,3,5-tribromo-2,4,6-trifluorobenzene), Merck KGaA [hexafluorobenzene, $Ir(ppy)_3$] and Carbolution Chemicals GmbH $Pd(PPh_3)_4$, Silicycle (SiO_2, particle size 40–63 µm). 4,4,5,5-Tetramethyl-2-(bicyclo[2.2.1]heptadien-2-yl)-1,3,2-dioxaborolane (**1e**) [2] and $Ru(phen)_3(PF_6)_2$ [3] were prepared according to literature. *n*-Hexane was purified by distillation prior to use.

4. Absorption properties

Solutions (c = 20 µM, V = 3.00 mL) of the norbornadiene **1f** in MeCN, cyclohexane, MeOH, EtOAc, THF, CH_2Cl_2, $CHCl_3$, and benzene were prepared by the evaporation of a stock solution of **1f** in MeCN (c = 1 mM, V = 60 µL) under a nitrogen stream followed by the addition of the corresponding solvent. The absorption spectra were subsequently measured (20 °C).

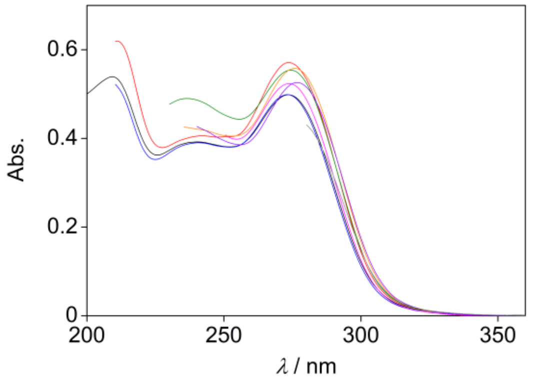

Figure S1: Absorption spectra of **1f** (c = 20 µM) in MeCN (—), cyclohexane (—), MeOH (—), EtOAc (—), THF (—), CH_2Cl_2 (—), $CHCl_3$ (—) and benzene (—).

5. Photoreactions

Photometric investigation of the cycloaddition and cycloreversion reaction

The cycloaddition reaction was investigated photometrically by the direct irradiation (λ = 315 nm) of **1f** in MeCN (c = 20 µM, V = 3.00 mL). The irradiation was stopped, when only negligible change in absorption occurred.

NMR-Spectroscopic investigation of the cycloaddition and cycloreversion reaction

A solution of **1f** (c = 6 mM, C_6D_6) was irradiated with LUMOS 43 (λ = 315 nm), and the product mixture was analyzed with ^{1}H NMR spectroscopy (Figure S11).

The photocatalyzed isomerization of **1f** (c = 15 mM, C_6D_6) in the presence of $Ir(ppy)_3$ (10 mol %) or the flavin **4** (10 mol %) was followed by in-situ ^{1}H and ^{19}F NMR spectroscopy (λ = 405 nm, 8.0 mW or 2.1 mW optical energy).

6. Radical-cation-induced cycloreversion

Magic blue (0.15 mg, 7 mol %) was added to a solution of the quadricyclane **2f^{0,3}** (c = 2 mM, $CDCl_3$). The subsequently recorded ^{1}H NMR spectrum showed complete conversion to the norbornadiene **1f** (Figure S12).

7. Kinetic studies of the thermal back conversion

The thermally induced cycloreversion of **2f^{0,3}** was monitored by absorption spectroscopy (Figure S2). A solution of the norbornadiene (c = 20 µM, V = 3 mL, isooctane) was irradiated for 3.5 h with λ_{ex} = 315 nm to give the corresponding solution of the quadricyclane **2f^{0,3}**. The increasing absorption ($\lambda_{abs.}$ = 274 nm) of the formed norbornadiene was determined at 70 °C, 80 °C, and 90 °C.

Considering that the cycloreversion is a monomolecular reaction, first-order reaction kinetics were applied for further analysis (Eq. 1).

$$-\frac{\mathrm{d[QC]}}{\mathrm{d}t}=\frac{\mathrm{d[NBD]}}{\mathrm{d}t}=k \Leftrightarrow \mathrm{[NBD]}_t=\mathrm{[NBD]}_{t=0}\,\mathrm{e}^{-kt} \qquad \text{(Eq. 1)}$$

In equation 1, [QC] is the concentration of the quadricyclane **2f^{0,3}**, [NBD] is the concentration of the norbornadiene **1f** and t is the time. The constant k describes the rate constant of the cycloreversion at a certain temperature and was obtained by an exponential fit.

With the rate constants k at their distinct temperature, ln(k/T) was plotted versus $1/T$ in an Eyring plot. The activation enthalpy ΔH was determined from the slope, while the entropy ΔS was determined from the y-intercept. The rate constant k for T = 25 °C was determined according to Eq 3.

$$\ln\left(\frac{k}{T}\right)=\frac{-\Delta H^{\ddagger}}{R}\,\frac{1}{T}+\ln\left(\frac{k_\mathrm{B}}{h}\right)+\frac{\Delta S^{\ddagger}}{R} \qquad \text{(Eq. 2)}$$

$$k=\frac{k_\mathrm{B}T}{h}\mathrm{e}^{-\frac{\Delta H^{\ddagger}-T\Delta S^{\ddagger}}{RT}} \qquad \text{(Eq. 3)}$$

In equations 2 and 3, k_B is the Boltzmann constant, T is the temperature, h is the Planck constant and R is the gas constant.

With the temperature-adjusted rate constant *k*, the half-life of the quadricyclane **2f^{0,3}** was determined (Eq. 4).

$$t_{1/2}=\frac{\ln(2)}{k} \qquad \text{(Eq. 4)}$$

In equation 4, $t_{1/2}$ is the half-life and *k* is the rate constant of the cycloreversion at T = 25 °C.

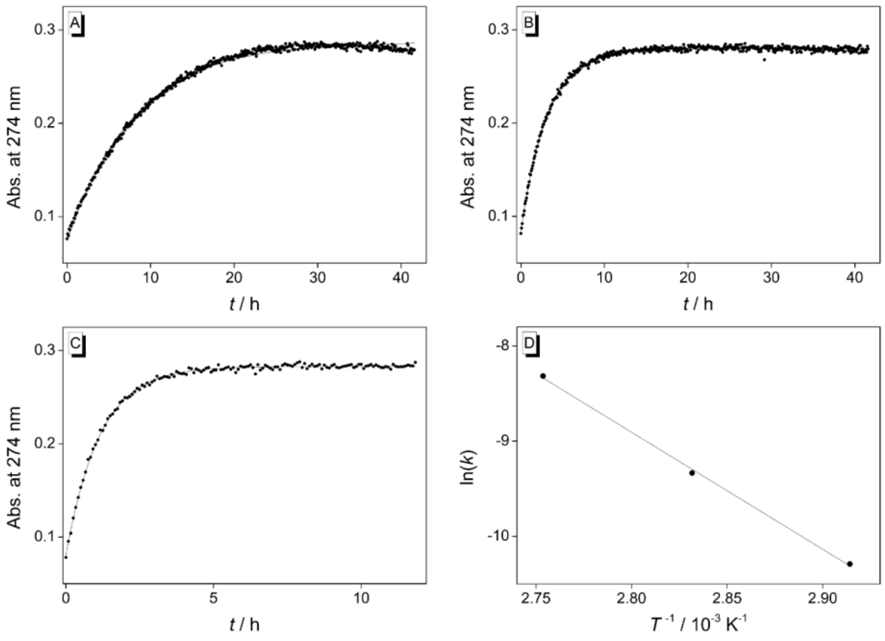

Figure S2: Photometric analysis of the thermal back reaction of **2f^{0,3}** (c = 20 µM, V = 3 mL) in isooctane at 70 °C (A), 80 °C (B), and 90 °C (C). The line represents the fit of the exponential function (A–C). The straight line represents the fit in the Eyring plot (D).

8. STA measurement

The quadricyclane **2f^{0,3}** (8.03 mg) was sealed in a platinum crucible with a pierced lid and placed in the calorimeter at room temperature under a nitrogen-gas atmosphere. The following temperature program was used to measure the heat release [4].

1. Maintain 25 °C for 15 min.
2. Heat from 25 °C to 250 °C with 5.0 °C min^{-1}.
3. Cool to 70 °C with 5.0 °C min^{-1}.
4. Heat from 70 °C to 250 °C with 5.0 °C min^{-1}.
5. Cool to 25 °C with 5.0 °C min^{-1}.

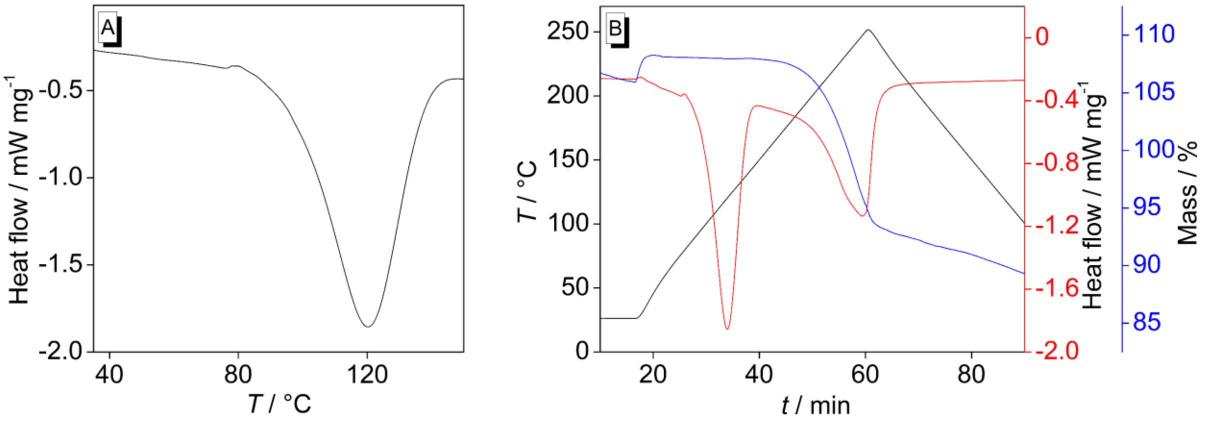

Figure S3: Extracted thermogram (A) from the STA measurement (B) of the quadricyclane **2f**^{0,3}. Negative signals of the heat flow represent exothermic processes, while positive signals represent endothermic processes (A, B). In the STA measurement (B) the heating rate (—), heat flow (—), and mass (—) are represented.

9. NMR spectra

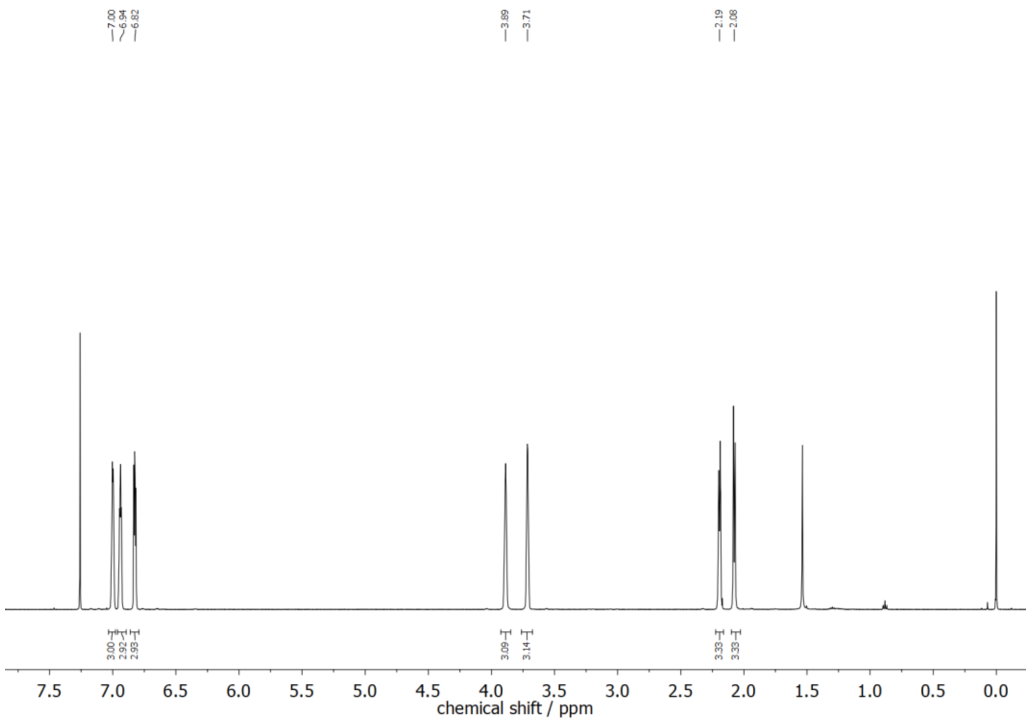

Figure S4: ^{1}H NMR spectrum (500 MHz) of **1f** in $CDCl_3$.

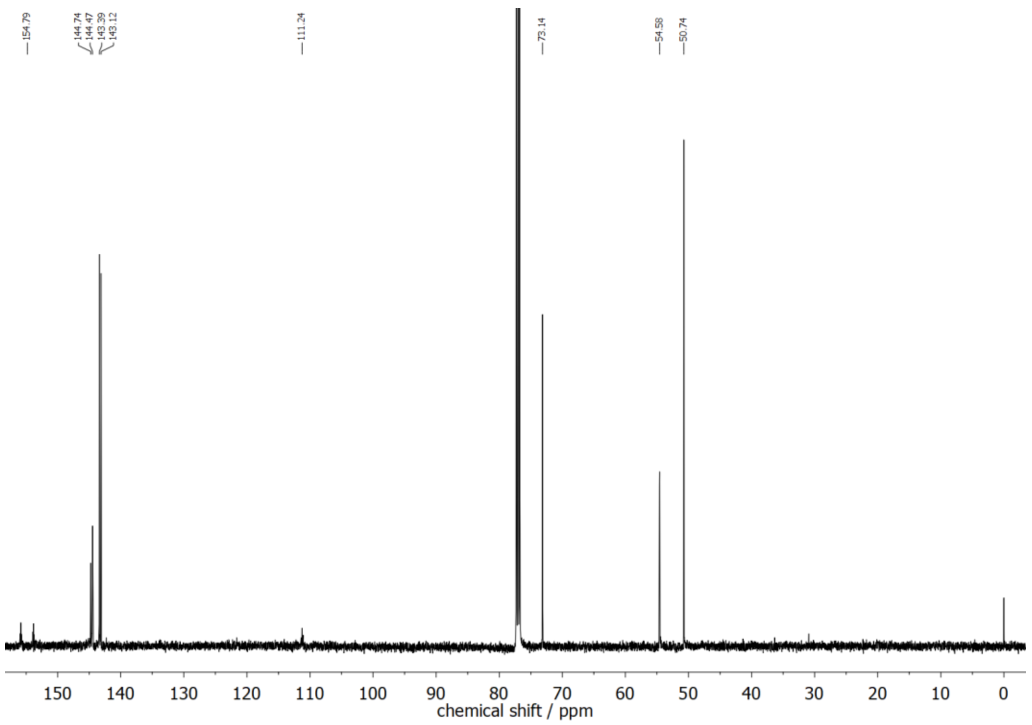

Figure S5: ^{13}C NMR spectrum (125 MHz) of **1f** in $CDCl_3$.

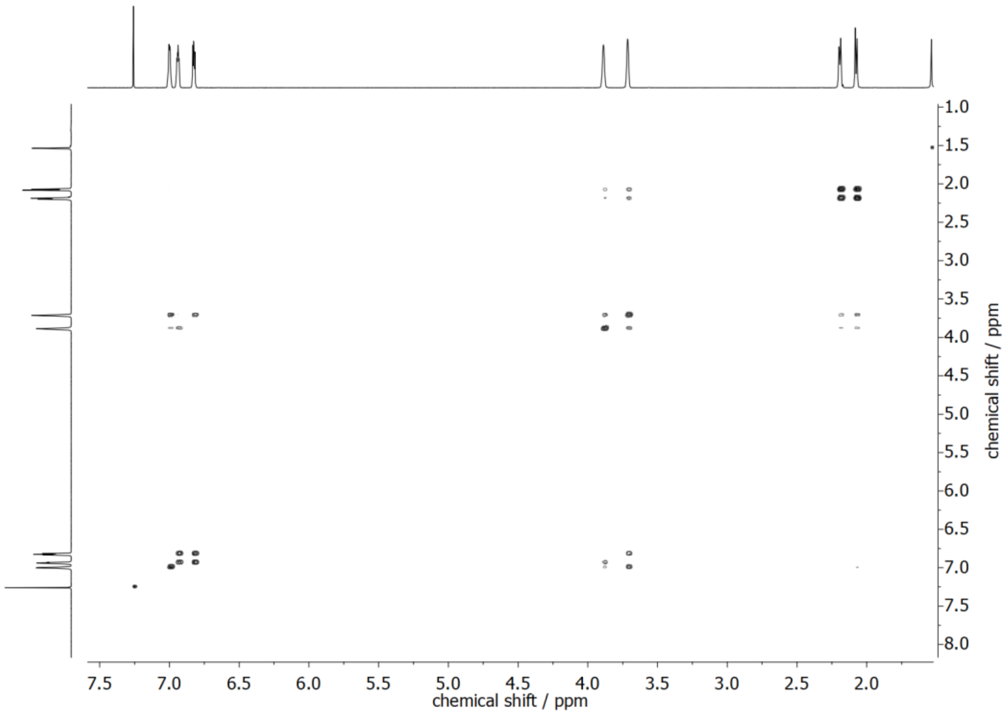

Figure S6: $^{1}H,^{1}H$-COSY NMR spectrum (500 MHz) of **1f** in $CDCl_3$.

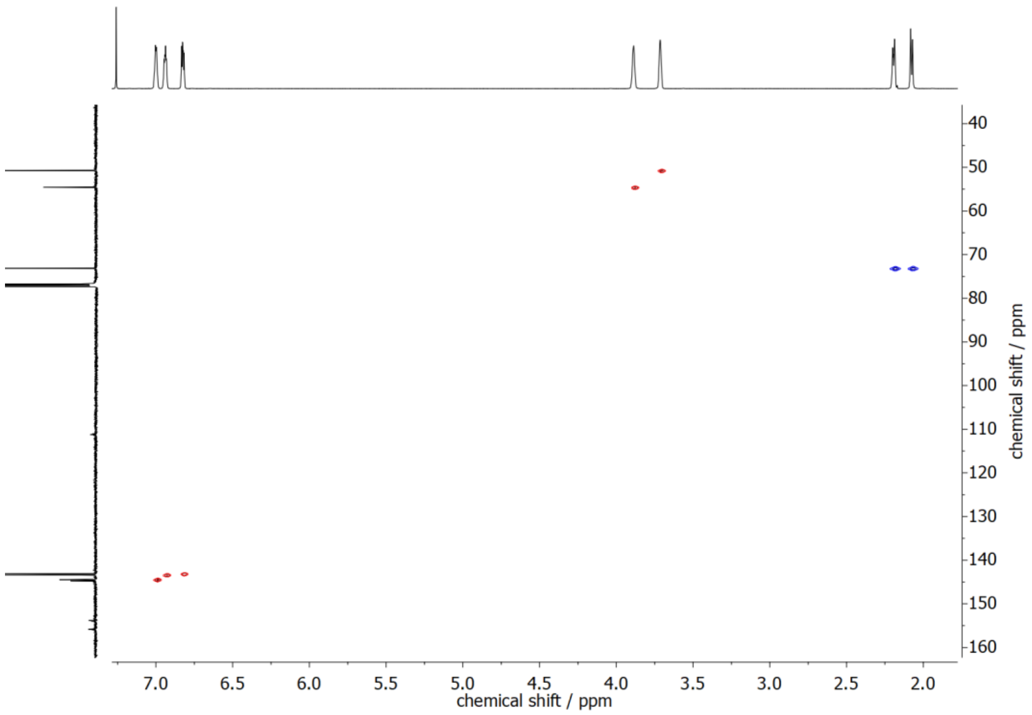

Figure S7: HSQC NMR spectrum (500 MHz) **1f** in $CDCl_3$.

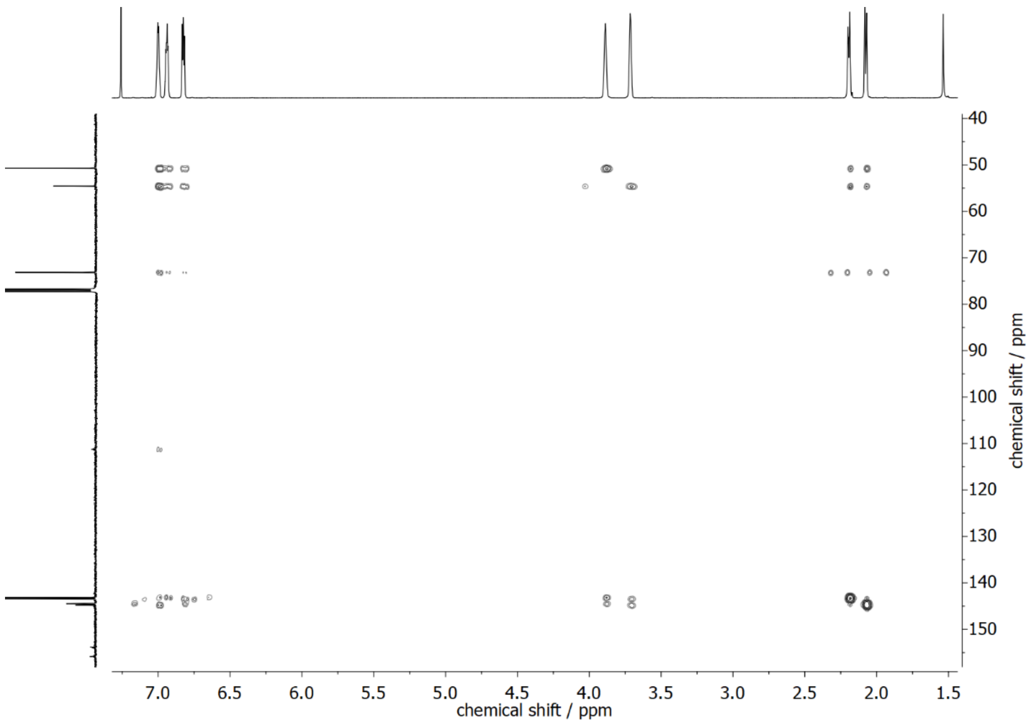

Figure S8: HMBC NMR spectrum (500 MHz) of **1f** in $CDCl_3$.

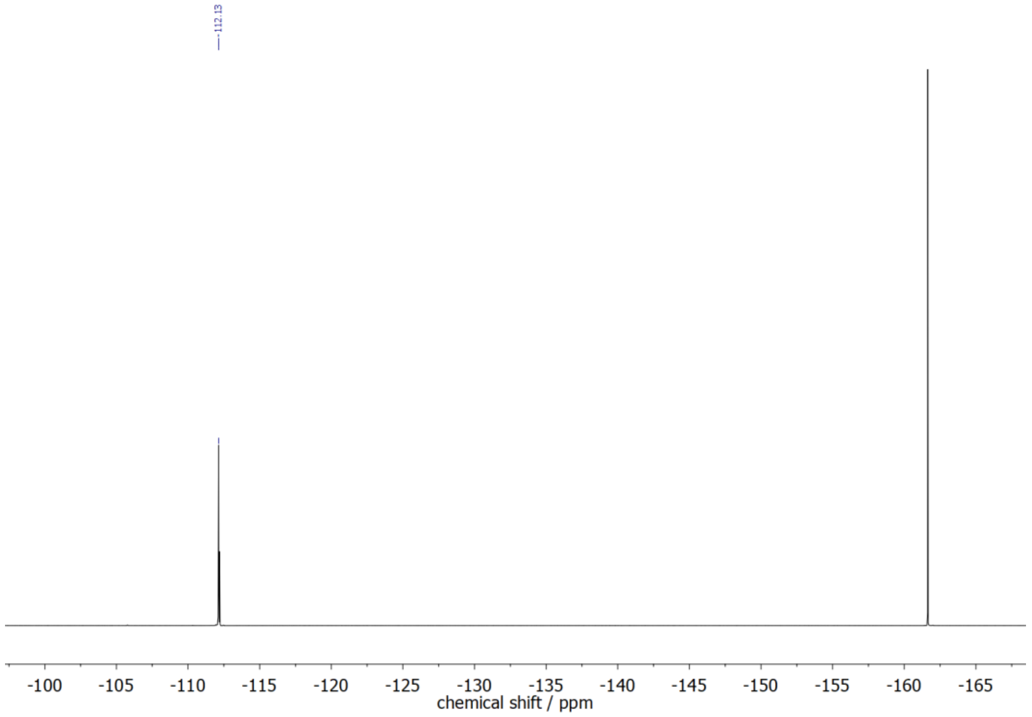

Figure S9: ^{19}F NMR spectrum (470 MHz) of **1f** in $CDCl_3$.

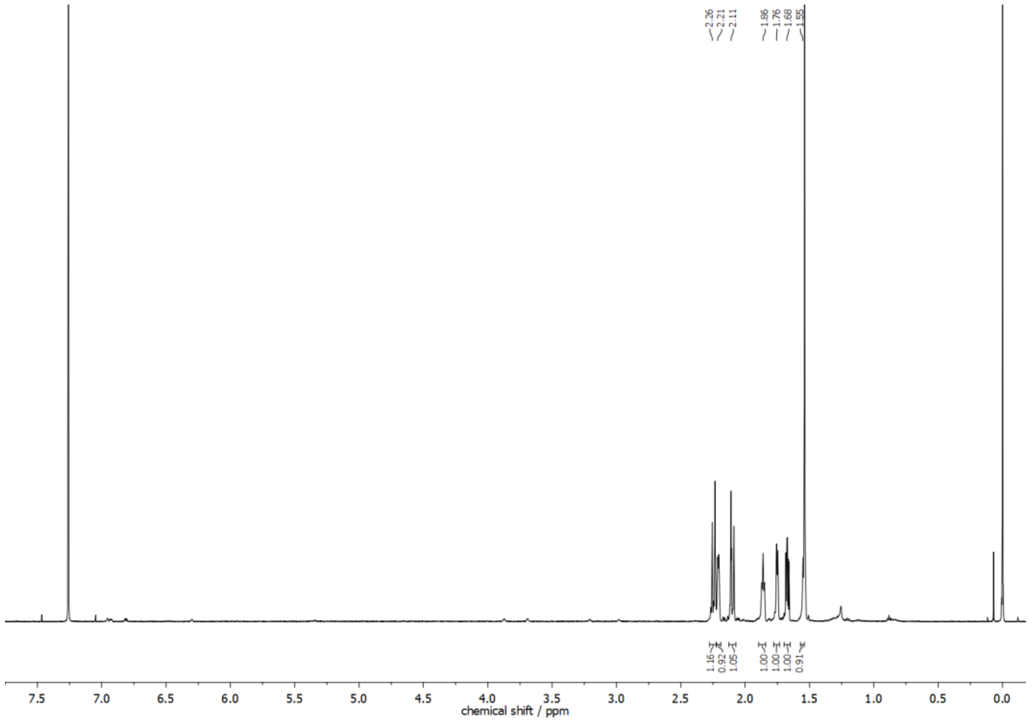

Figure S10: ^{1}H NMR spectrum (500 MHz) of $\mathbf{2f^{0,3}}$ in $CDCl_3$.

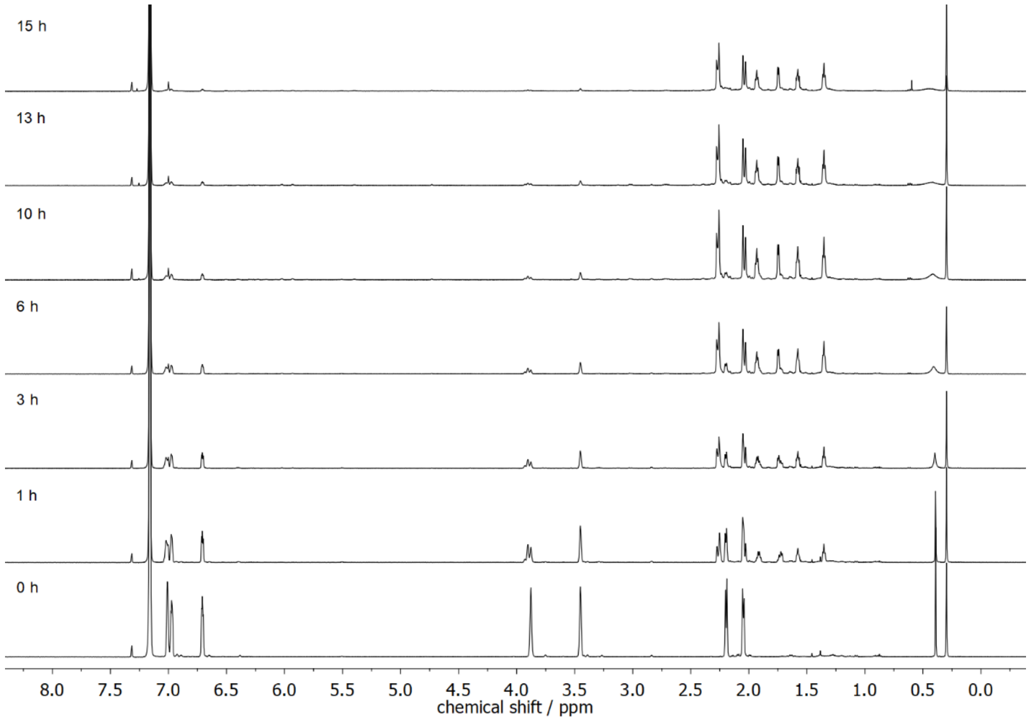

Figure S11: Determination of the photostationary state (PSS) by monitoring the cycloaddition reaction of **1f** (c = 6 mM) in C_6D_6 upon irradiation with LUMOS 43 (λ_{ex} = 315 nm) with ^{1}H NMR spectroscopy (500 MHz).

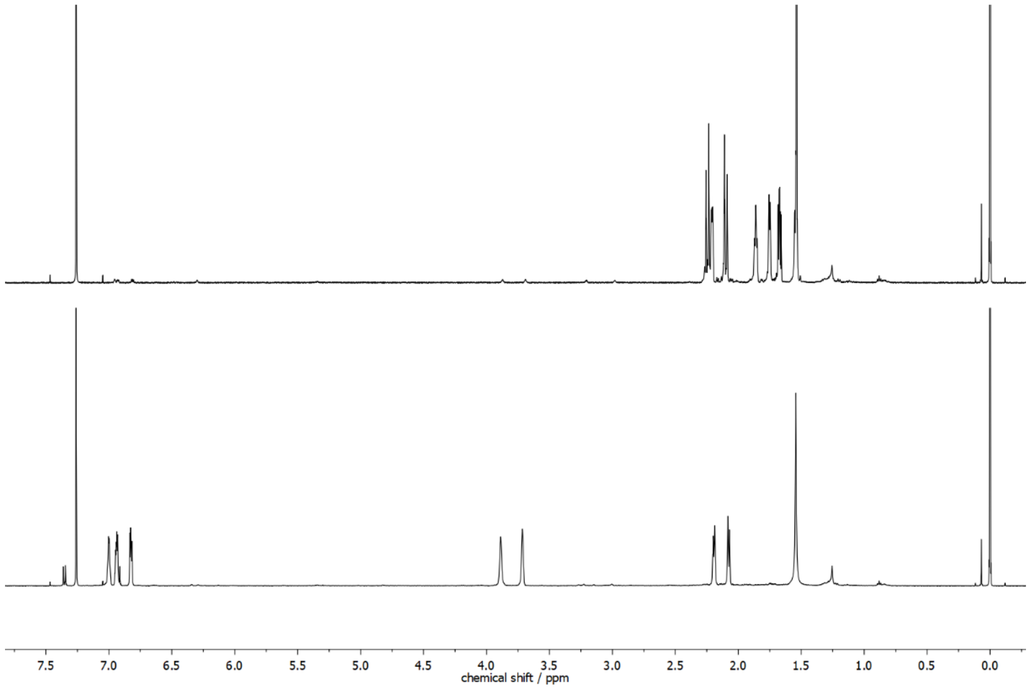

Figure S12: ^{1}H NMR spectra (500 MHz) of **2f^{0,3}** (c = 2 mM, $CDCl_3$, top spectrum) and after addition of magic blue (7.5 mol %, bottom spectrum)

10. In situ NMR spectroscopy

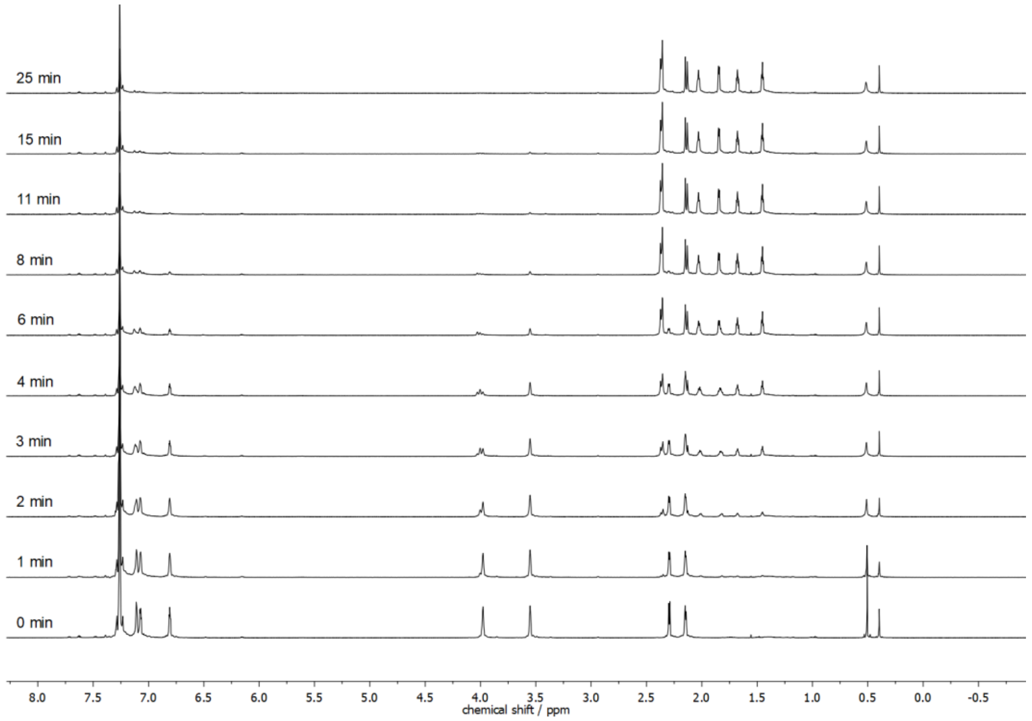

Figure S13: In situ ^{1}H NMR-spectroscopic analysis (600 MHz) of the photoreaction of **1f** (15 mM) in the presence of $Ir(ppy)_3$ (10 mol %) in C_6D_6 at different irradiation times (λ_{ex} = 405 nm, 8.0 mW). Spectra were recorded in intervals of 1 min, only selected spectra are shown.

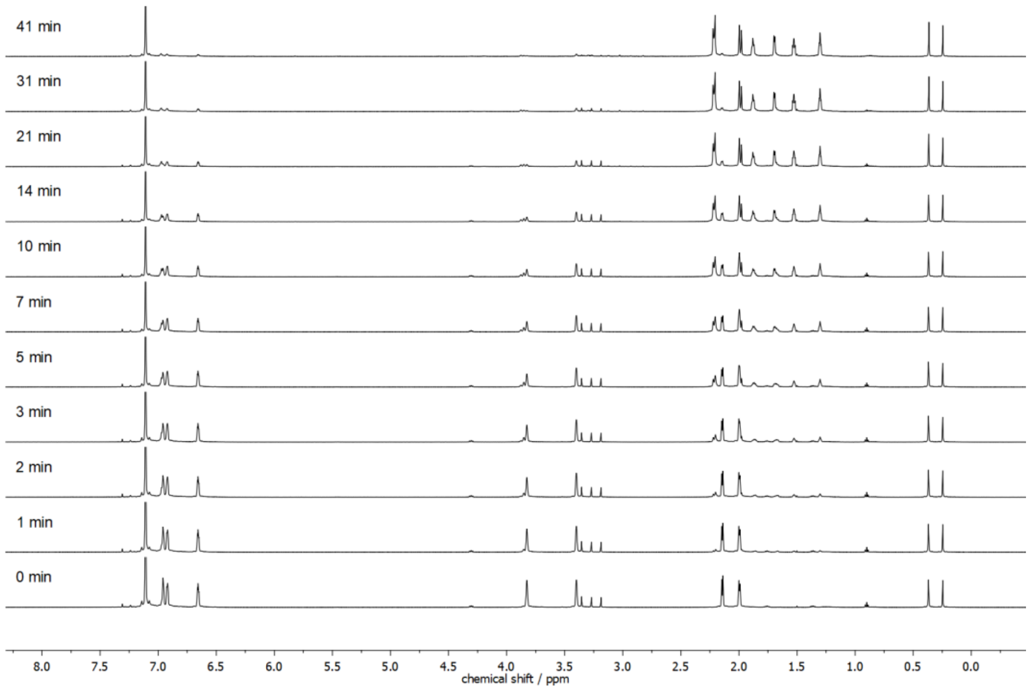

Figure S14: In situ ^{1}H NMR-spectroscopic analysis (600 MHz) of the photoreaction of **1f** (15 mM) in the presence of flavine **4** (10 mol %) in C_6D_6 at different irradiation times (λ_{ex} = 405 nm, 2.1 mW). Spectra were recorded in intervals of 1 min, only selected spectra are shown.

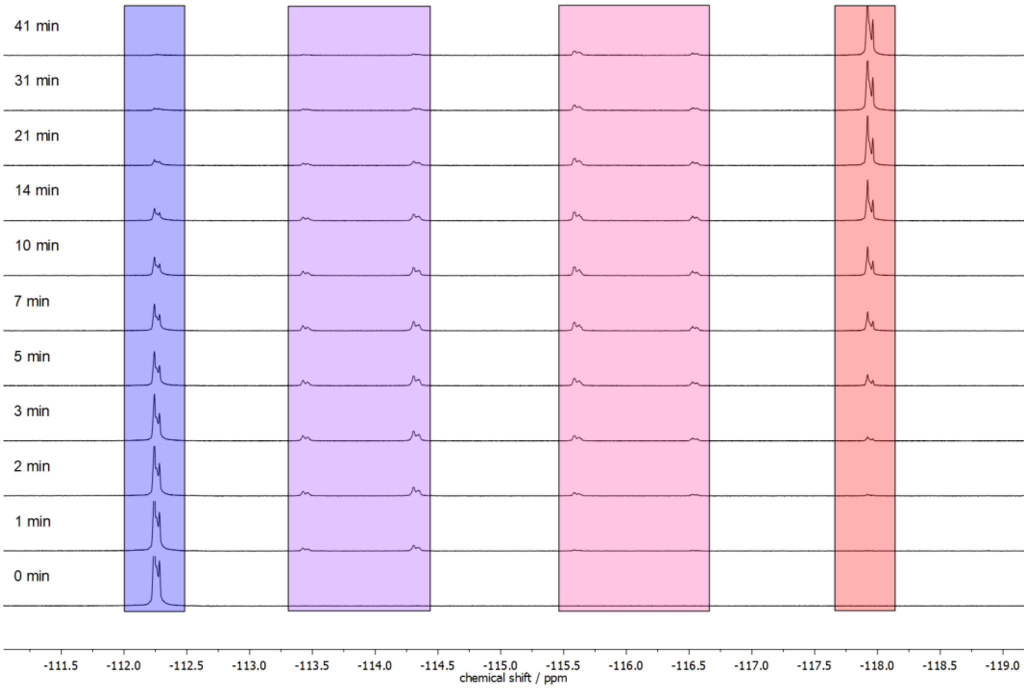

Figure S15: In situ ^{19}F NMR spectroscopic analysis (565 MHz) of the photoreaction of **1f** (15 mM) in the presence of flavine (**4**) (10 mol %) in C_6D_6 at different irradiation times (λ_{ex} = 405 nm, 2.1 mW). The blue band indicates the signals of **1f**, the purple band indicates the signals of **2f**^{2,1}, the pink band indicates the signals of **2f**^{1,2}, and the red band indicates the signals of **2f**^{0,3}. Spectra were recorded in intervals of 1 min, only selected spectra are shown.

11. Kinetic simulation

The rate constants (k_1, k_2, k_3) of the photosensitized cycloaddition reaction of **1f** were estimated by fitting the change of concentrations of the starting material **1f**, photoproducts **2f**^{2,1}, **2f**^{1,2}, and **2f**^{0,3} and photocatalyst **4** during reaction time. For this purpose, the Complex PAthway SImulator COPASI [5] was used in combination with the Levenberg–Marquardt algorithm. The concentrations were obtained by integration of the ^1H and ^{19}F signals as examined by in situ NMR-spectroscopic analysis (Figure S14 and S15).
It was assumed that the reaction is a stepwise and irreversible process (Eq. 5, 6, 7). The ground state and excited state of the photocatalyst were not explicitly considered, but rather treated as a collision factor. Hence, three reactions were used for the analysis (Eq.5–7).

$$\mathbf{1f} + \mathbf{4} \xrightarrow{k_1} \mathbf{2f}^{2,1} + \mathbf{4} \qquad \text{(Eq. 5)}$$

$$\mathbf{2f}^{2,1} + \mathbf{4} \xrightarrow{k_2} \mathbf{2f}^{1,2} + \mathbf{4} \qquad \text{(Eq. 6)}$$

$$\mathbf{2f}^{1,2} + \mathbf{4} \xrightarrow{k_3} \mathbf{2f}^{0,3} + \mathbf{4} \qquad \text{(Eq. 7)}$$

In equation 5, 6, 7, k_1, k_2, and k_3 represent the respective rate constants of these reactions.

Over the course of the reaction photobleaching occurred (Figure S14) and was considered in the fitting according to (Eq. 8).

$$\lim_{t\to\infty} [\mathbf{4}]t \xrightarrow{k_{bleach}} 0 \qquad \text{(Eq. 8)}$$

In equation 8, [**4**] is the concentration of the photocatalyst, t is the time and k_{bleach} the rate constant of the bleaching process.

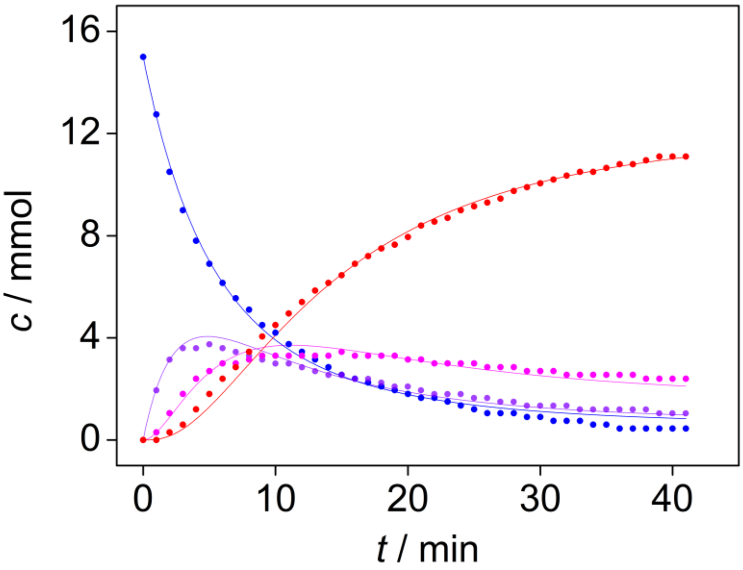

Figure S16: Plot of the concentration of components in the reaction mixture versus reaction time of **1f** in the presence of **4** (λ_{ex} = 405 nm); blue: **1f**; purple: **2f^{2,1}**; pink: **2f^{1,2}**; red: **2f^{0,3}**. The solid lines represent the corresponding fit of the experimental data to the kinetic model.

Table S1: Rate constants k_1, k_2, and k_3 of the stepwise photoreaction of trisnorbornadiene **1f**.

	k / L mmol^{-1} min^{-1} [a]	SD / 10^{-3} L mmol^{-1} min^{-1}
k_1	0.12	1.0
k_2	0.20	2.6
k_3	0.17	2.2

[a] Rate constant obtained from fit of experimental data to kinetic model (eq. 5–7).
[b] Standard deviation from parameter estimation.

12. References

1. Paululat, T.; Rabe, M.; Berdnikova, D. V. *J. Magn. Reson.*, **2021**, *327*, 106990.
2. Schulte, R.; Ihmels, H. *Beilstein J. Org. Chem.*, **2022**, *18*, 368–373.
3. Wang, W.; Zhang, J.; Wang, H.; Chen, L.; Bian, Z. *Appl. Catal. Gen.*, **2016**, *520*, 1–6.
4. Schulte, R.; Afflerbach, S.; Paululat, T.; Ihmels, H. *Angew. Chem. Int. Ed.*, **2023**, *62*, e202309544.
5. Hoops, S.; Sahle, S.; Gauges, R.; Lee, C.; Pahle, J.; Simus, N.; Singhal, M.; Xu, L.; Mendes, P.; Kummer, U. *Bioinformatics*, **2006**, *22*, 3067–3074.
